# biogrowleR: Enhancing Longitudinal Data Analysis

**DOI:** 10.1007/s10911-025-09583-7

**Published:** 2025-06-03

**Authors:** Carlos Ronchi, Giovanna Ambrosini, Flavia Hughes, Renée L. Flaherty, Hazel M. Quinn, Daria Matvienko, Andrea Agnoletto, Cathrin Brisken

**Affiliations:** 1 School of Life Sciences, ISREC - Swiss Institute for Experimental Cancer Research, Ecole Polytechnique Fédérale de Lausanne (EPFL), Lausanne, CH-1015 Switzerland; 2 The Breast Cancer Now Toby Robins Breast Cancer Research Centre, The Institute of Cancer Research, London, UK; 3 Bioinformatics Competence Center, Ecole Polytechnique Fédérale de Lausanne, Lausanne, 1015 Switzerland; 4 Bioinformatics Competence Center, Université de Lausanne, Lausanne, 1015 Switzerland

## Abstract

Time course measurements are used for many applications in biomedical research, ranging from growth curves to drug efficacy testing and high-throughput screening. Statistical methods used to analyze the resulting longitudinal data, such as t-tests or repeated measures ANOVA have limitations when groups are unbalanced, or individual measurements are missing.

To address these issues we developed biogrowleR (https://upbri.gitlab.io/biogrowleR/), a workflow to visualize and analyze data based on Frequentist and Bayesian inference combined with hierarchical modeling. By focusing on effect sizes we enhance data interpretation. The workflow further includes a randomization algorithm important to reduce numbers of experimental animals (RRR) and costs. The workflow and R package were designed to be used by researchers with limited experience in R and biostatistics.

Our open-source R package biogrowleR contains tutorials, pipelines, and helper functions for the analysis of longitudinal data and enables non computational scientists to perform more effective data analysis.

## Background

**Fig. 1:**
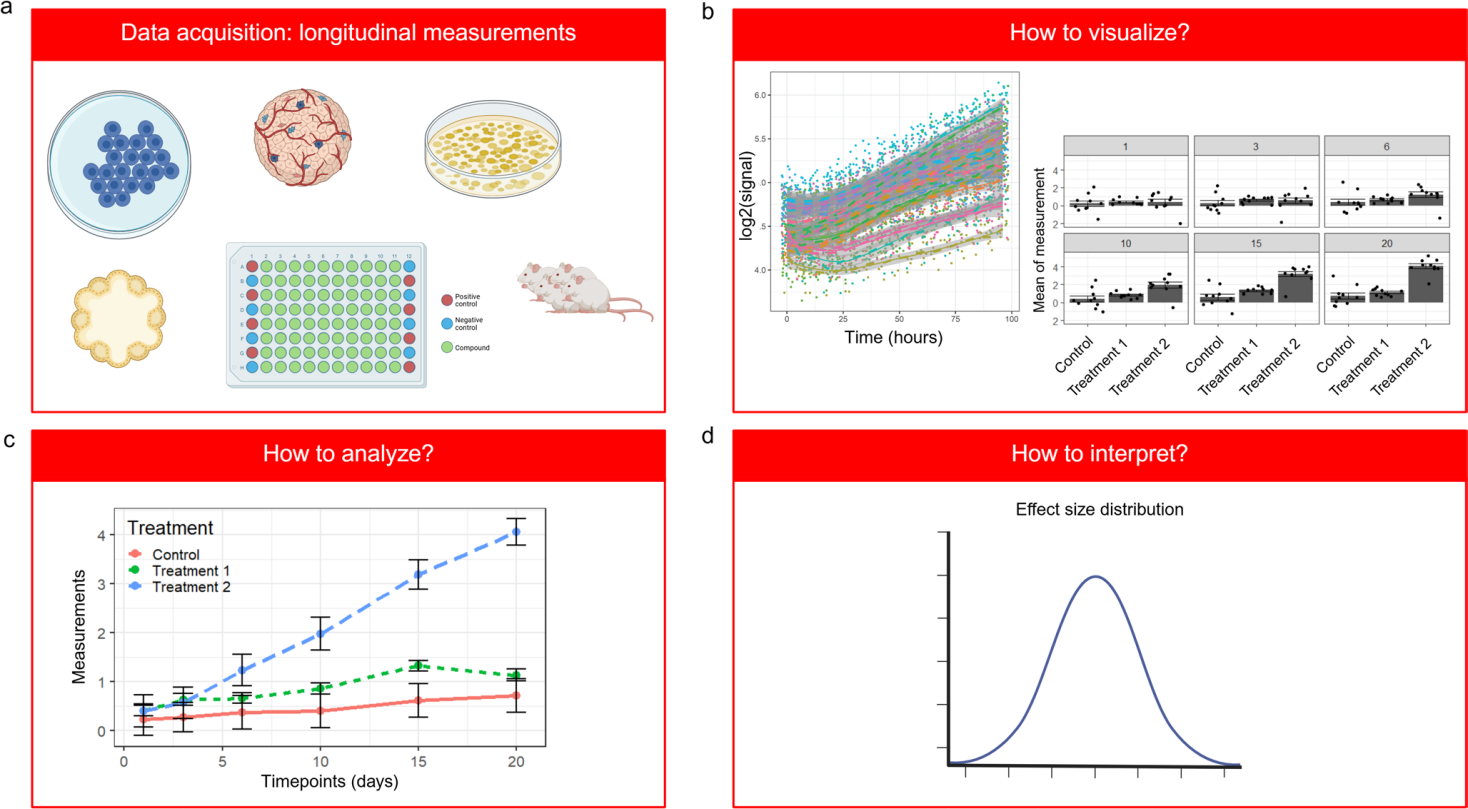
Longitudinal measurements: flowchart. Panels representing the steps in longitudinal data analysis: **a** data acquisition, **b** data visualization, **c** data modelling and **d** interpretation of results

Many research activities in biology and biomedical sciences rely on longitudinal data acquisition (Fig. 1a). Examples are in vitro or in vivo cell growth curves, in vivo tumor growth, drug screens and genetic perturbation assays. Measurements can range from seconds, hours to months resulting in data of similar structure which pose common challenges for analysis. Typically, the large number of data points makes data visualization and exploration difficult (Fig. 1b). Choosing the appropriate statistical method (Fig. 1c) and interpretation of the results – in particular understanding of the magnitude of effect size can be challenging (Fig. 1d). Often results are statistically significant but have a small effect size and are hence not biologically meaningful on their own.

Data exploration, a crucial step in any analysis pipeline, includes identification of outliers and of the distribution of variables. When sequential measurements for a single sample are acquired, it is important to understand not only the effect at endpoint, but also the relationship of the different datapoints over time. This relationship can be reflected by a simple linear relationship, or a more complex one that can be modeled using advanced functions such as polynomials or splines with pre-specified degrees and knots [1, 2].

When common statistical tools such as t-tests are applied to each timepoint they inflate Type I error, i.e., the rejection of the null hypothesis when it is true [3]. More advanced statistical methods, such as repeated measures ANOVA or MANOVA, rely on a balanced study design and require that there be no missing measures over time [4]. These prerequisites can be difficult to meet, especially when performing in vivo experiments with multiple variables that are difficult to control. In tumor xenograft experiments, f.i., take rates vary, and varying in engraftment rates may result in different tumor sizes. Multiple measurements may be missing either for technical reasons or due to animal welfare issues. Some measurements rely on the detection of a reporter such as luciferase and improper injection of the tracer, luciferin, may affect the signal detected.

Hierarchical modeling, also called mixed effects model, has been used to analyze data with repeated and missing measurements [3–8]. Although hierarchical modeling is not commonly employed by biologists and non-statisticians, when they do use it, the effect sizes are often not fully explored. Typically, the magnitude and direction (positive or negative) of the effect do not significantly influence their final interpretation of the results.

Here we integrate Bayesian inference within a hierarchical modeling framework, particularly suited for analyzing complex longitudinal in vivo measurements such as tumor growth over time. While traditional frequentist methods rely heavily on assumptions like balanced designs, no missing data, and interpretation based on *p*-values, the Bayesian approach addresses these limitations more naturally and informatively.

Bayesian hierarchical models provide a more nuanced and robust framework for longitudinal data analysis for various reasons. First of all they can incorporate prior knowledge. Information from earlier experiments like prior distributions is particularly useful when data are sparse timepoints or small cohorts. This helps in particular when dealing with variable growth rates. A further advantage is the better handling of missing data. Bayesian models naturally accommodate missing values under the assumption that data are missing at random. This is particularly important for in vivo measurements, where practical or ethical issues may arise. Finally, Bayesian inference provides a full posterior distribution for each parameter rather than just a point estimate. This allows the researcher to directly assess the probability that an effect is positive or exceeds a biologically meaningful threshold. By focusing on the posterior distribution of the effect size rather than the significance tests, researchers are encouraged to consider biological relevance rather than merely statistical significance. This shift aligns with contemporary best practices in biomedical research.

As such, we have taken a more holistic approach using established statistical tools. Differences between experimental conditions are interpreted using effect sizes and taking into account the dynamics of the obtained longitudinal data. To facilitate interpretation of the results and maximize the use of research resources, we use both Bayesian and frequentist paradigms to derive effect sizes and propose better standards for longitudinal data analysis done by bioscientists.

A new algorithm ensures sample groups are well randomized based on multiple pretreatment timepoints. The approach is illustrated with two types of real data: an in vivo tumor growth experiment and an in vitro drug screening experiment. The R package, called biogrowleR (https://upbri.gitlab.io/biogrowleR/), is completed by tutorials and helper functions to guide the user through the analysis step by step.

## biogrowleR Workflow

### Data Exploration and Visualization

As an example of biogrowleR capabilities and illustrating the workflow, in this and the next section, we will use data on in vivo growth of a patient-derived xenograft (PDX) using bioluminescence measurements. The data were generated from slow growing estrogen receptor positive breast cancer samples using the intraductal xenografting approach (MIND) [9, 10]. These tumors grow slowly and the resulting data are challenging to analyze, as measurements are often amiss and the experimental groups are unbalanced. Estrogen receptor-positive (ER+) breast cancer is a hormone-driven disease treated with targeted therapies. Steroid hormones, such as estrogen and progesterone affect tumor cell proliferation. In order to better model ER + BC in vivo and understand the effects of estradiol (E2), progesterone (P4) and their combination on the tumor cells, PDXs were generated using the MIND approach. For this, tumor cells were engineered to express the Green Fluorescent Protein-luciferase (GFP-Luc) reporter. Luciferase is an enzyme that when in presence with the luciferin substrate leads to a light-emitting reaction, referred to as bioluminescence. Mice were then injected with patient derived GFP-Luc expressing tumor cells into the ducts of one or multiple mammary glands. Tumor cells expressing luciferase were tracked in the animals in vivo thanks to the cell bioluminescence in the presence of luciferin. When the average luminescence reached a defined threshold, mice were treated with vehicle, E2, P4 or combined E2 + P4 for 64 days by means of slow-release drug pellets implanted subcutaneously [10]. Thus, bioluminescence emanating from each individual mammary gland could be measured, and differences in signal over time and across the treatment conditions could be compared.

**Fig. 2:**
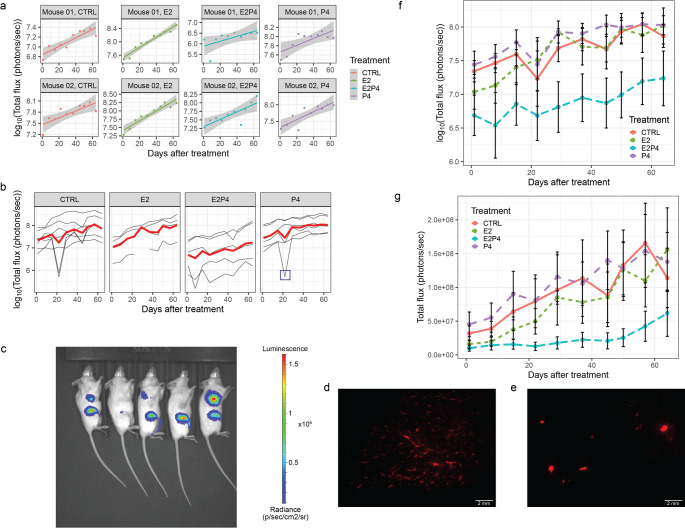
Steps of the exploratory data analysis. **a** Luminescence curves for each mouse and measurements taken. The color corresponds to the different treatment given to the mice for 64 days. **b** All growth curves for all glands plotted and stratified by condition. The curve in red corresponds to the average of all curves in the specified condition. The blue square in the P4 plot highlights an outlier measurement of one of the growth curves. **c** Representative IVIS image from mice with injected glands **d** Representative fluorescence stereomicrograph of an inguinal mammary gland in which the intraductally injected RFP expressing tumor cells spread within the mouse milk ducts. **e** Representative fluorescence stereomicrograph of an injected mammary gland where RFP expressing tumor cells have invaded into stroma the ducts. **f** Growth curves summarized in log_10_ or **g** Graph showing Total Flux (photons/second) over time of treatment. Error bars represent standard error

The visualization tools are readily available on R or any other programming language and recommendations are provided. The first step is to explore the data and to identify outliers. Outliers occur when expression of the GFP-luciferase reporter is shut down or the injection fails. Individual time plots can be viewed for each sample, i.e. each individual mouse mammary gland injected with labelled tumor cells (Fig. 2a). Such plots reveal different types of patterns of bioluminescence measurements over time and inform the modelling of the data. When plotting the individual growth curves together, stratified by different treatments, some control (CTRL) and P4 treated samples show a sharp drop in a single measurement around day 20 (Fig. 2b). This is likely attributable to an unsuccessful luciferin injection and could be considered as an outlier measurement. Raw images obtained from the in vivo imaging system (IVIS) measurements (Fig. 2c) may show samples without detectable signal. Stereomicroscopy complements IVIS images and reveals the distribution of fluorescence emitted by the GFP reporter in the mammary gland ducts. A well injected sample has fluorescence signal distributed all over the mammary gland reflecting cells that spread well within the milk ducts (Fig. 2d). On the other hand, a badly injected sample typically shows individual large foci, attributable to cells that are not well spread but growing outside the ducts (Fig. 2e).

The ultimate goal of this type of analysis is to identify changes in total flux (photons/second) relative to a control condition. To this end, a summary growth curve shows the $\:{\text{l}\text{o}\text{g}}_{10}$-scale total flux (Fig. 2f) and the total flux (Fig. 2g), for each condition. These curves illustrate some sources of variability and the influence of each measurement on the mean. For example, the measurements around day 20 for the CTRL samples were significant enough to lower the mean. Error bars highlight the extent of variability. Overall, in the present example there are no samples that can be considered outliers, so no measurements need to be discarded.

### Modeling the Data Generating Process

Each gland with injected tumor cells in a mouse corresponds to an experimental unit. Up to four glands in the same mouse are routinely injected. This experimental unit is the first level of nesting in the data since measurements are taken sequentially for each gland and so a timepoint is not independent from the previous one. Glands are further stratified by mice, being the second level of nesting. Mice can be housed in different cages, leading to a third level of nesting. Next, each mouse is assigned to a specific treatment, in this case the different hormone treatments. This is a covariate of interest. Another covariate of interest is time, i.e., the time at which the measurements were taken. To conclude, the $\:{\text{l}\text{o}\text{g}}_{10}$ total flux is modeled as a function of time and treatment covariates as fixed effects, and the repeated measurements for a single gland are included as a grouping variable in the hierarchical model. Equation 1 represents the R formula used to estimate the effects of time and treatment considering repeated measurements.

1 $$\begin{aligned}\:{\text{l}\text{o}\text{g}}_{10}\left(\text{T}\text{o}\text{t}\text{a}\text{l}\:\text{F}\text{l}\text{u}\text{x}\left(\frac{\text{p}\text{h}\text{o}\text{t}\text{o}\text{n}\text{s}}{\text{s}\text{e}\text{c}}\right)\right)=&\;\text{t}\text{i}\text{m}\text{e}+\text{t}\text{r}\text{e}\text{a}\text{t}\text{m}\text{e}\text{n}\text{t}+\text{t}\text{i}\text{m}\text{e}\\&\times\text{t}\text{r}\text{e}\text{a}\text{t}\text{m}\text{e}\text{n}\text{t}+\left(1|\text{i}\text{d}\right)\end{aligned}$$

Each condition has a different starting average total flux (Fig. 2a and b) captured by $\:\left(1\:\right|\:id)$ in the formula (Eq. 1). Equation (1) is independent of the statistical framework used and both the frequentist or the Bayesian approach can be applied as described below. This proposed approach is called hierarchical modelling, described in more detail below, or mixed/random effects models.

#### Bayesian Framework

The Bayesian framework focuses on the interpretation of the results based on the *posterior distribution* of estimated parameters from the data. The posterior distribution is proportional to the likelihood of the collected data multiplied by the prior information available (Eq. (2), Bayes theorem), for example past events or previous experiments.

2 $$\:\text{P}\text{o}\text{s}\text{t}\text{e}\text{r}\text{i}\text{o}\text{r}\propto\:\text{l}\text{i}\text{k}\text{e}\text{l}\text{i}\text{h}\text{o}\text{o}\text{d}\:\times\:\text{p}\text{r}\text{i}\text{o}\text{r}$$

The posterior distribution can also be expressed in terms of condition probabilities (Eq. (3)):

3 $$\:\text{P}\left(\text{A}|\text{B}\right)=\frac{\text{P}\left(\text{B}|\text{A}\right)\text{P}\left(\text{A}\right)}{\text{P}\left(\text{B}\right)}$$

where A and B are events and P(B) is different from 0. The probability of event A happening given event B (P(A|B)) is a conditional probability also called the posterior probability. The P(B|A) is the probability of the event B given A, or likelihood of the data if A corresponds to the data and B are the parameters being estimated when performing Bayesian inference. P(A) and P(B) correspond to the prior information without any conditions. Since parameters are distributions, probabilistic arguments can be made.

**Fig. 3:**
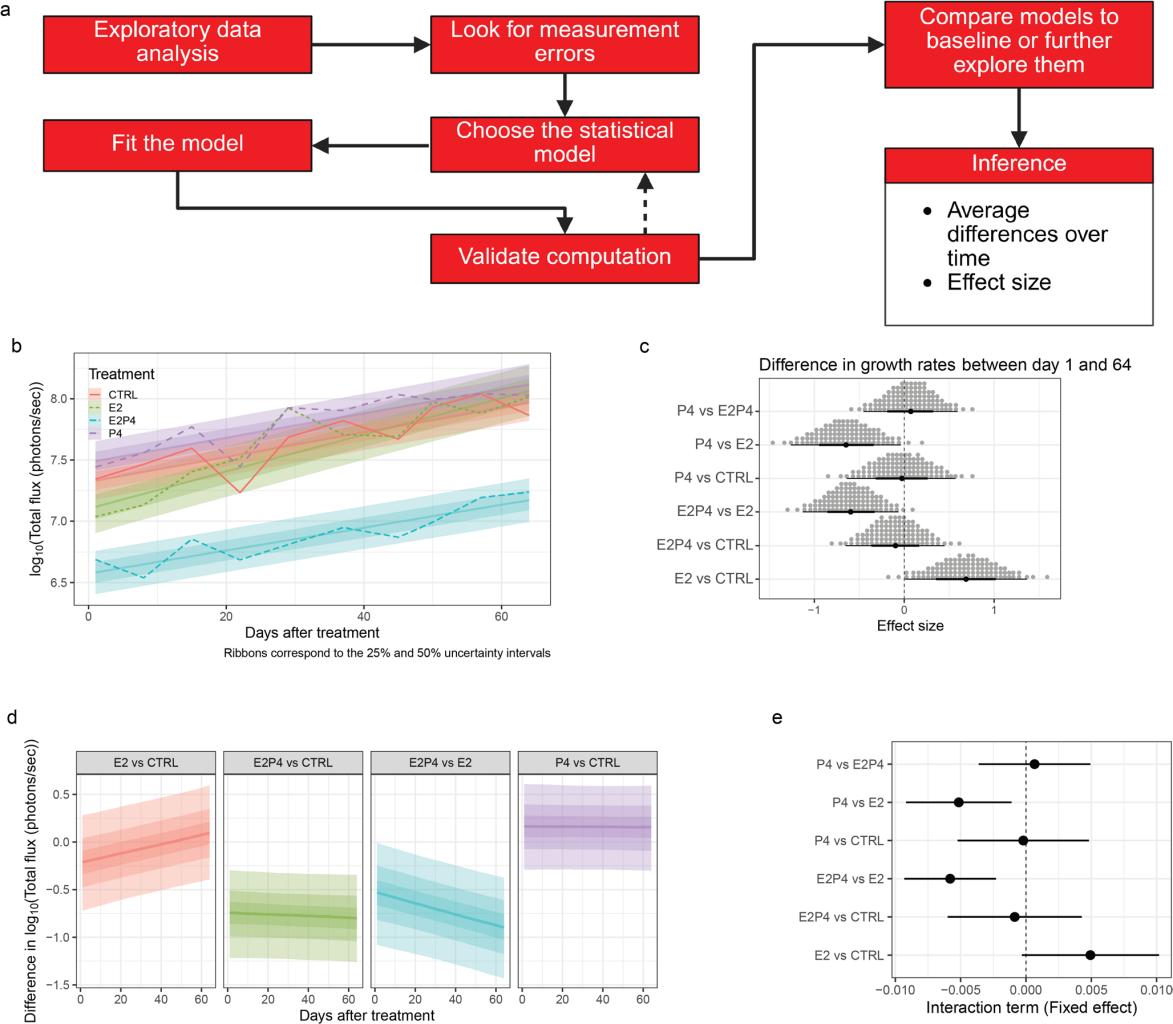
Modeling and interpreting the results. **a** Scheme showing the proposed workflow comprising data generation process and interpretation of the results. **b** Growth curves with overlapping best-fit line estimates from a hierarchical linear regression using each individual measurement with varying intercept. The different shades of the interval represent the 25% and 50% uncertainty intervals obtained from the posterior distribution. **c** Dot plots showing the distribution of effect sizes for each individual treatment comparison. Each dot corresponds to a 1% quantile of the expected posterior distribution. The effect size was calculated as the difference between the slopes of the estimated lines using all the time points. **d** Differences of the predicted growth curves when compared either to control or E2. CTRL: control, E2: 17-β-estradiol, P4: progesterone, E2P4: 17-β-estradiol plus progesterone. **e** Interaction terms obtained when using the frequentist approach for all pairwise comparisons

Assuming that the model captures the data generating process, different probabilistic arguments can be used to explain the effect size. In our example, the bioluminescence signals of each PDX over time are modeled by linear regression using the mouse as the grouping variable (corresponding to each individual unit of measurement). In this way, growth rate estimates are obtained for each treatment (Fig. 3b) and comparisons between conditions can be made. One of the differences between Bayesian and frequentist frameworks concerns the uncertainty intervals. On the one hand, in the frequentist framework, the confidence interval assumes that data can be sampled an infinite number of times and the true estimate, in this case the growth rate, will be within the confidence interval 95% of the time. On the other hand, the uncertainty interval associated with estimates obtained by the Bayesian approach represents the credible interval. Bayesian estimates are based on the *posterior distribution*, which describes the uncertainty about statistical parameters conditional on a collection of observed data. In the example, based on the linear regression estimates, growth rates can be compared across conditions (see Methods) (Fig. 3c). In this context, the initial question “is there any difference between the conditions” can be resolved by rephrasing it to “what is the probability that the effect size is greater than 0”. This is different from the frequentist point of view that depends on a null hypothesis. Since we are modelling the data generating process, there is no null hypothesis. Figure 3c shows the effect size distributions for each pairwise comparison of interest. Each dot corresponds to a 1% quantile of the *posterior distribution* of the difference in growth rates. Thus, to answer the initial question, we have to determine the probability that the effect size is greater than 0. It is necessary to count the number of dots that exceed 0. When comparing E2 versus CTRL, 98 of the 100 dots are above 0 indicating a 98% probability this is the case. There is no established probability threshold for considering a significant effect because the question can always be reformulated, and the probability will change in each case. The next step is to determine whether there is any difference between conditions of interest. Rather than tackling this problem directly, it may be more useful to understand the magnitude and direction of the differences. This is done by modeling the data generating process through the use of statistical models (Fig. 3a). After choosing the correct statistical model, or the one closest to the data generating process, the model needs to be validated through convergence checks and other methods [1]. Problems in model validation can result from misspecification, i.e., the model does not match the data generating process, there is an insufficient number of data points, or from too many outliers. Once a valid model is obtained, it can be compared with a baseline model and inferences can be made, and the effect size can be determined. In the case of the growth curves different features can be analyzed; first, the difference between the slopes of the growth curves when modeling the data with linear regression and corresponds to the interaction term, second the difference between the initial and final values for two different conditions. The last option does not depend on linear models and polynomial or splines can be used to model the data.

It is also important to consider the growth kinetics and/or the shape of the curves to understand to what extent conditions, in our case hormone treatments, increase or decrease growth. Figure 3d shows an alternative way of looking at the difference between some of the pairwise comparisons of interest. The direction of estimated growth gives an indication of the differences between the conditions of interest. The different starting points of the curves show that some of the conditions had a higher or lower total flux at the beginning of the experiment. Informative priors are encouraged in order to stabilize the parameter estimation (see Methods: Hierarchical Modelling). For example, in the particular MIND-PDX experiments, the prior distribution is a normal distribution with mean 7 and a standard deviation of 2. The mean 7 corresponds to an average value of $\:lo{g}_{10}$ total flux usually used as a threshold to start a drug treatment in engrafted tumor experiments [9, 10].

#### Frequentist Framework

According to the frequentist approach, the likelihood ratio test can be used to compare different models and thereby to determine whether growth rates differ between conditions. The idea is to have one full model that includes the interaction between time and treatment and another model in which this interaction is not present. The interaction estimates the differences between growth rates of different treatments and assumes the difference between the growth rates of the treatments is equal to 0 (null hypothesis), while the alternative hypothesis is the difference is not 0. To evaluate the null hypothesis we use the *lrtest* function from the R package *lmtest* to obtain a final *p*-value. If the question of interest concerns only two treatments, the models must be fitted to a subset of the data containing only the conditions of interest. Again, the difference between the fitted lines can be calculated. Confidence intervals for the estimated differences are calculated using bootstrapping [11].

In our example, Table 1 shows the results of the different statistical tests performed. The results obtained are similar to the Bayesian ones. Only the interpretation is different. With a significance level $\:{\upalpha\:}=0.05$, the null hypothesis for E2 vs. CTRL and E2 vs. E2 + P4 is rejected with E2 having higher growth rates in both comparisons. The interaction term and its 95% confidence interval can also be visualized (Fig. 3e). The interaction term corresponds to the difference in growth rate without any adjustment for initial variance at the beginning of the treatment. To summarize, these are the two ways to analyze and interpret growth measurement data once the data generating process is modeled.

**Table 1:** Results of the likelihood ratio test (LRT) for each pairwise comparison are given in the *Comparison* column. The *p-value* column indicates the *p*-value obtained from the LRT and the associated test statistics are provided in the *Statistics* column

Comparison	*p*-value	Statistics
CTRL vs. E2	6.78E-02	3.33E + 00
CTRL vs. E2P4	7.41E-01	1.09E-01
CTRL vs. P4	9.36E-01	6.47E-03
E2 vs. E2P4	1.69E-03	9.86E + 00
E2 vs. P4	1.41E-02	6.02E + 00
E2P4 vs. P4	7.63E-01	9.10E-02

### Sample Randomization

When using in vivo models, it is important to understand growth kinetics to inform the appropriate starting point for any treatments. Overall growth kinetics should be considered when assigning mice to the different treatment cohorts due to inherent variability. Longitudinal measurements are acquired prior to treatment to use for randomization. We developed a new algorithm to randomize mice into different groups, considering the growth kinetics or the shape of the growth and, if necessary, blocking factors, such as caging, to avoid further bias.

**Fig. 4:**
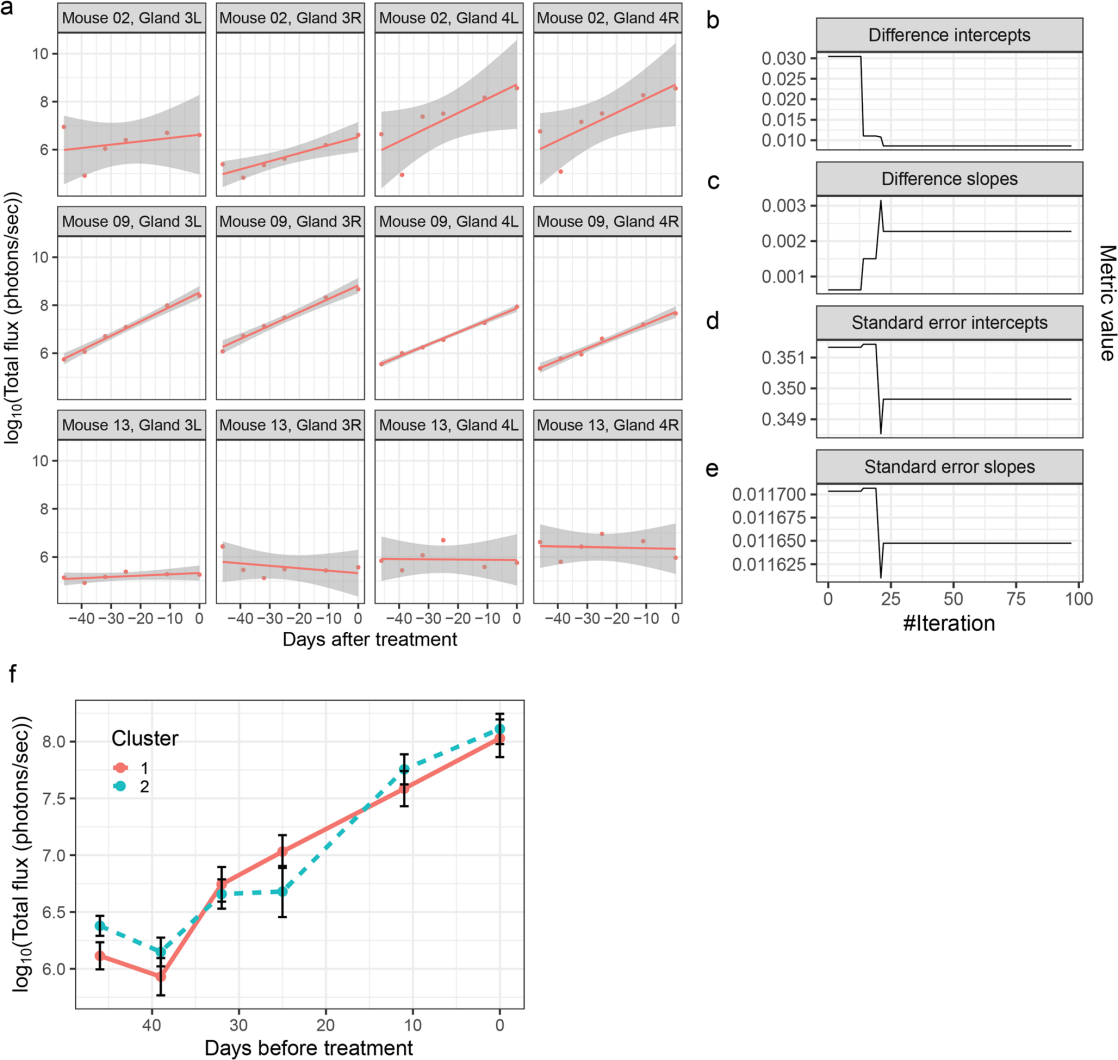
Randomizing and defining groups. Steps in the randomization process. **a** Plots showing in vivo luminescence of individual xenografted glands of three host mice prior to treatment. **b**-**e** Metrics used for randomization as a function of the total number of iterations used by the randomization algorithm. Four different metrics are used: i) pairwise difference of the average endpoint measurements for the expected group, ii) pairwise difference of the slopes for the assigned groups in each iteration, and respectively the same for iii) standard error of the intercept and iv) the standard error of the slopes. **f** Average growth curves for the two groups assigned by the randomization algorithm. The x-axis shows days before treatment in decreasing order

First, data are explored to detect outliers (Fig. 4a). All 4 injected glands of mouse 13 show constant signal, while luminescence increases exponentially with time in all other injected glands. This suggests that the tumor cells did not engraft well in the glands in mouse 13. As such, these glands need to be excluded from randomization to prevent skewing of the data.

Subsequently, the retained measurements are randomized. In the case of in vivo tumor measurements, there may be multiple measurements per mouse. Therefore, it is important to make sure that in a single mouse the measurements are not highly variable. In this case, it is recommended to remove the entire experimental unit from the randomization process so as not to distort the grouping. Randomization is then performed at the level of individual mice. In the present example, we randomize 8 mice into two groups, named *cluster 1* and *2*. The method seeks to minimize the sum of pairwise differences of: growth rates, estimated averages of last luminescence measurements, standard deviation of growth rate, and standard deviation of the estimated average of last luminescence measurements between the groups. Since pairwise differences are considered, the number of groups is limited only by the number of available experimental units. By default, the *get_best_splitting* function runs for 100 iterations minimizing the mentioned metrics (see Methods: Simulated annealing). “Diagnostic plots” allow to check convergence of the minimization process and to control successful randomization. The convergence curves (Fig. 4b-e) show the value of the respective metrics in each iteration. As a general rule, the sum of pairwise differences at the last measurement point and of the growth rates should decrease with iterations while the sum of pairwise differences between standard deviations should be stable. An acceptable situation is a decrease in the summed pairwise difference between the last measurement point of the groups (Fig. 4b) and increase in the summed pairwise difference of the growth rates (Fig. 4c). The final grouping can be extracted from the results of the *get_best_splitting* function, and the growth curves can be visualized using the default functions in *biogrowleR* (Fig. 4f).

Following randomization it is important to ensure that groups are balanced with regards to other factors that may influence long-term measurements like cages and weight. Our algorithm includes a way to randomize mice into groups by blocking any categorical factors.

## Screening Assay Analysis

**Fig. 5:**
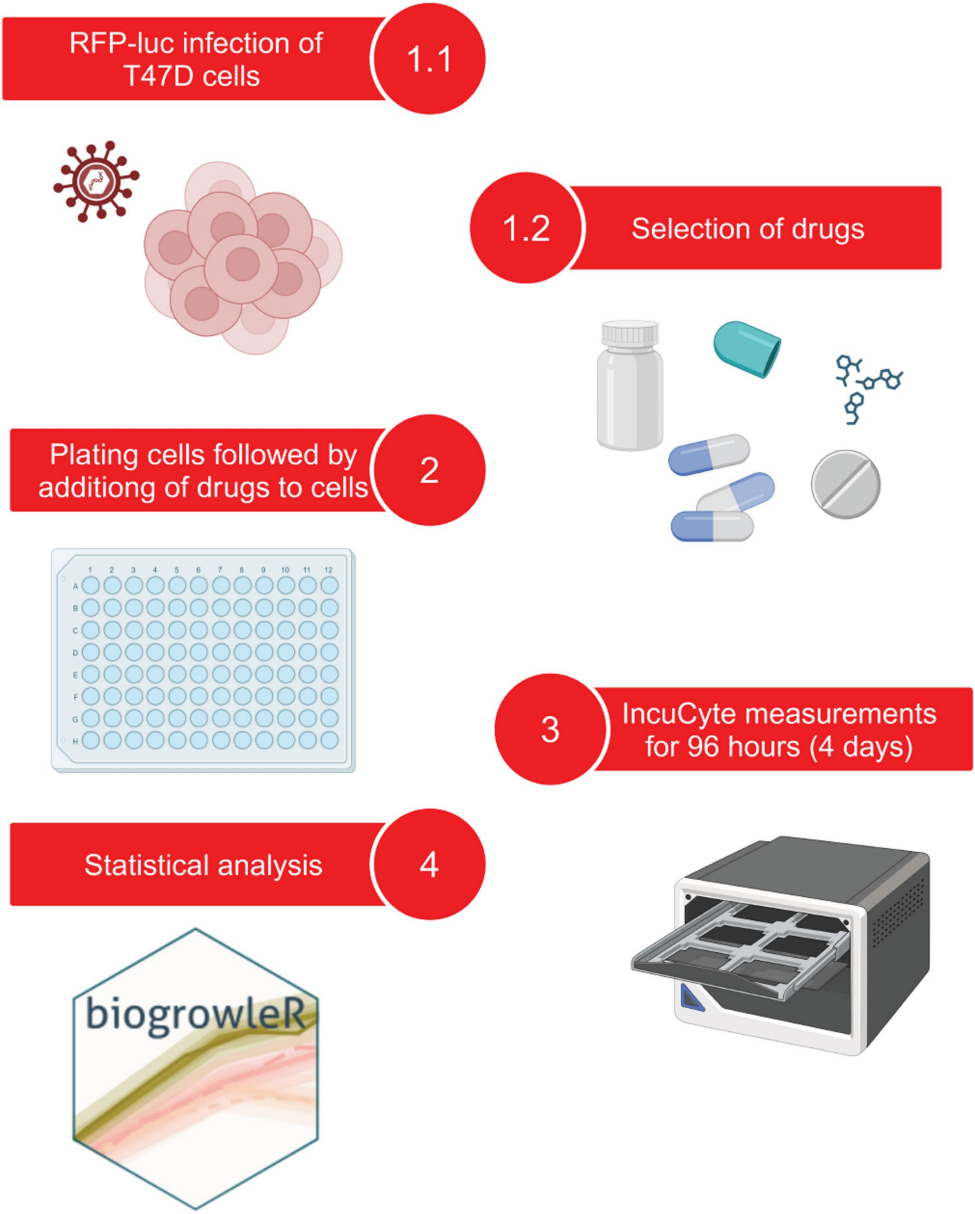
Workflow representing the generation of high-throughput drug screening. First step is the infection of T47D cells with RFP-Luc2, selected drugs are added to cells plated in a 96-well-plate. Incucyte measurements are taken every 6 hours for 4 days and statistical analysis of the results is performed using the *biogrowleR* R package

Screening assays usually involve multiple conditions (drugs, genetic perturbations) and combinations thereof and use different readouts. These types of experiments, often with longitudinal measures, generate large amounts of data that pose a challenge for their analysis. Here, we propose a pipeline using Bayesian inference to interpret this type of data. We perform a drug screening assay in a 96-well plate format with the estrogen receptor ER and progesterone receptor positive breast cancer cell line, T47D engineered to express *RFP-Luc2 *[9]. Cells were treated with single agents or combinations and confluency/fluorescence measured over 4 days at 6 h intervals using Incucyte SX3 (See Methods; Fig. 5). A total of 25 treatment conditions were used with 12 replicates per condition.

**Fig. 6:**
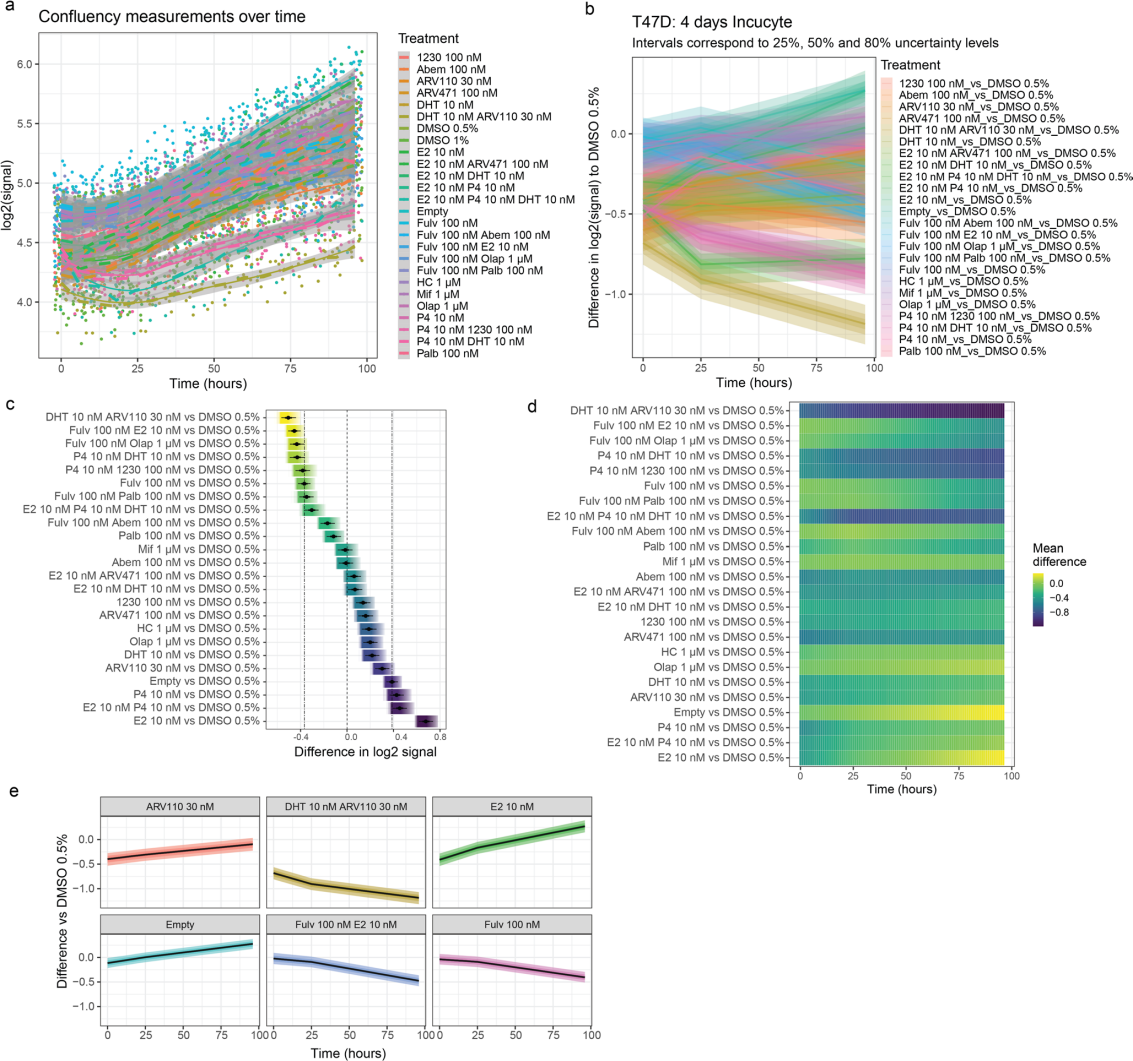
Drug screening analysis. **a** Luminescence signal of cells embedded in hydrogel as a function of time. Twenty-five conditions were measured at 8 timepoints over 96 h (*n* = 12). **b** Bayesian estimates of the data using linear splines with a single knot at t = 25h. The uncertainty intervals correspond to the 66% and 95% quantiles represented by the different shades. The lighter the color, the closer to the 95% quantile. **c** Forest plot showing differences at 96h compared to the control condition, with the gradient depicting the distribution of the difference. **d** Heatmap showing the average difference over time for all conditions compared to control. **e** Difference of the estimated growth curves compared to control. The black curve corresponds to the average difference compared to control over time. The uncertainty bands correspond to the 25%, 50%, 80% and 95% quantiles of the difference in each timepoint

When visualizing the data, if the number of conditions exceeds 4 it becomes difficult to distinguish individual ones (Fig. 6a). A possible solution is to draw only condition-specific curves, although this limits the complex nature of multiple drug screening.

The analysis pipeline traces parts of the previous in vivo image analysis. After examining the data, we attempt to model the data generating process as described earlier. For this dataset, we use splines to model the kinetics of the different curves. Graphical inspection of the data reveals a pronounced decrease in slope starting at 25 h, which prompts us to use a linear spline with a node at 25 h. After selecting the best model that includes a group variable for repeated measurements of each well, we plot the differences from the control (DMSO 0.5%, Fig. 6b). In this case, however, it is very difficult to tell which treatments are the best. Instead, we compare the end points of two conditions normalized to the initial time point:

$$\:\left({M}_{{t}_{1}}^{{c}_{1}}-{M}_{{t}_{1}}^{{c}_{0}}\right)-\left({M}_{{t}_{0}}^{{c}_{1}}-{M}_{{t}_{0}}^{{c}_{1}}\right),$$

where $\:{M}_{{t}_{i}}^{{c}_{i}}$ corresponds to the estimated $\:{\text{l}\text{o}\text{g}}_{2}$-scale measurement of the condition c_i_ at timepoint t_i_ for i = 0,1. In this way, we obtain an estimate of the fold-change with respect to a condition of interest and predetermined time points. Using a forest plot (Fig. 6c) of the fold changes for all treatments relative to the control (DMSO 0.5%) all results are captured in a single, simple plot. The graph is ordered according to increase in the average fold-change of different conditions over control. The gradients correspond to the 95% uncertainty intervals, i.e., the uncertainty of the Bayesian estimates. While the 95% reflects a widely used choice the uncertainty intervals can be calculated at any level. Another way to visualize differences between drugs is to show the average difference between condition and control at all time points using a heatmap (Fig. 6d). These strategies allow us to analyze large datasets comprehensively.

Once the conditions of interest have been identified from the initial screening, the kinetics of the drug effects can be analyzed. As an example, we select estimates of treatments with ARV110 (androgen receptor PROTAC), dihydrotestosterone (DHT) + ARV110, 17-b-estradiol (E2), Fulvestrant (Fulv), Fulv + E2 and no treatment (Empty) (Fig. 6e). On the one hand, Fulvestrant elicited a small decrease in the signal difference compared to DMSO 0.5% in the first 25 h followed by a steeper decrease, indicating a lag in response to Fulvestrant, consistent with its mechanism of action as an ER degrader. On the other hand, combining Fulvestrant with E2 does not affect Fulvestrant potency, even though E2 induces a positive difference in signal compared to DMSO 0.5%. ARV110 alone has a small stimulatory effect but inverts to show the largest decrease in signal of all treatments when combined with DHT, highlighting a potential synergistic effect between a receptor degrader (PROTAC) and a receptor agonist. In summary, we have demonstrated how the proposed workflow may be used to select effective drug strategies, and the scalable potential to identify top hits in drug screenings.

## Synthetic Longitudinal Data and Sensitivity Analysis

To better understand the data generation process and the modelling techniques used in our pipeline, we generate synthetic longitudinal data using the simstudy package [12] in R and assess how effect size and data variability influence the estimates obtained.

**Fig. 7:**
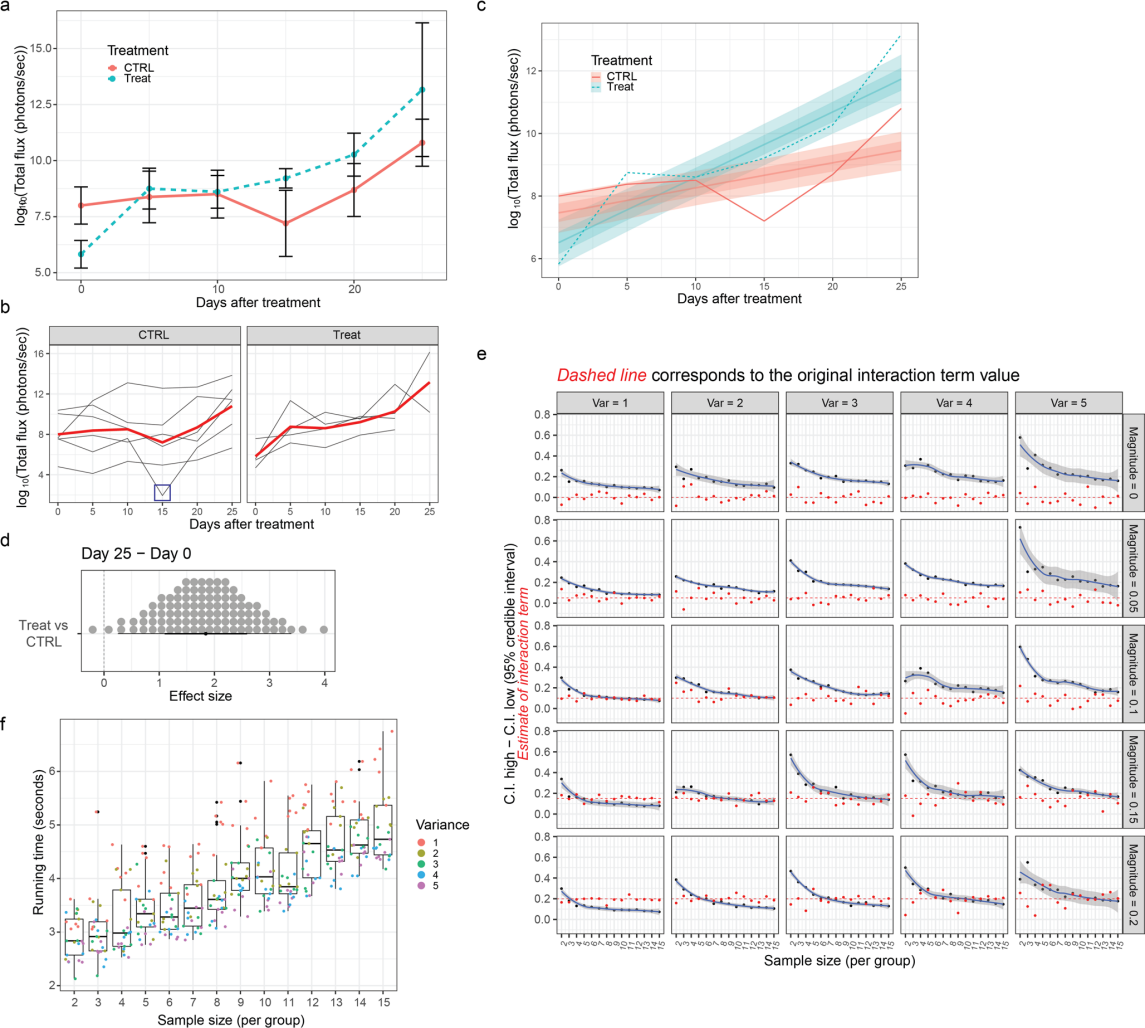
Synthetic longitudinal data and sensitivity analysis. **a** Summarized growth curves from a synthetic dataset (*n* = 6 control, *n* = 4 treatment). **b** All growth curves for all glands plotted and stratified by condition. The curve in red corresponds to the average of all curves in the specified condition. The blue square in the CTRL plot highlights an outlier measurement of one of the growth curves. **c** Bayesian estimates of the data. The uncertainty intervals correspond to the 66% and 95% quantiles represented by the different shades. **d** Dot plot showing the distribution of effect sizes for each individual treatment comparison. Each dot corresponds to a 1% quantile of the expected posterior distribution. The effect size was calculated as the difference between the slopes of the estimated lines using all the timepoints. **e** Difference in the extremes of the 95% credible interval of the estimated interaction term by sample size stratified by variance and magnitude of the interaction term. The red dots correspond to the estimated interaction terms between treatment arm and time given the variance and sample size. The dashed red line corresponds to the true interaction term. **f** Running time in seconds by sample size when running the *stan_glmer* from *rstanarm* with 4 chains, 2000 iterations and different variances

We first generate a dataset consisting of 6 control and 4 treated samples (Fig. 7a), i.e. we assign the treatment groups using a 50%/50% ratio for control and treatment respectively. The samples represent the mammary glands in our case. Visualization of individual curves stratified by treatment shows that the trend of the curves is similar to that of the IVIS data (Fig. 7b). All growth curves have different starting points and lack some data points. In addition, some measurements appear as outliers, such as a measurement on day 15 from the control group that drops below 4. Since we are simulating and knowing the data generating process, we can keep it. Otherwise an investigation of why this sample has a sudden drop in the measurement is necessary.

Next, the Bayesian effect size is calculated, as described in the previous section, using the entire dataset. The Bayesian estimates, where the uncertainty intervals correspond to the 66% and 95% credibilities, respectively, and are represented by different shades (Fig. 7c). These levels are chosen as they are used under the frequentist approach. We note that the slopes of control and treatment curves differ with an estimated interaction term about 0.1. The growth coefficient for the treatment group is overestimated, while for the control group it is correct. In fact, the credibility interval is very wide for the treatment estimate, which means that there is not enough data to accurately analyze the size of the treatment coefficient.

If we plot the distribution of effect sizes for each individual treatment/control comparison (Fig. 7d), where the effect size was calculated as the difference between the slopes of the estimated treatment and control lines respectively, using all time points, we see a positive effect size, confirming that the treatment induces growth compared to the control.

To understand the relationship between variance and sample size, given a fixed effect size, synthetic data was generated by changing the variance of the bioluminescence (1 to 5) and the number of samples per group (2 to 15) each time. The magnitude of the interaction term (or effect size) between time and treatment groups varied from 0 to 0.2 in increments of 0.05. As we said, the aim of this sensitive analysis was to be able to identify the minimum number of samples required to obtain a narrow credible interval and thus a good estimate of the effect size. A similar analysis could be done using confidence intervals under the frequentist paradigm. Under the Bayesian framework, priors could also be modified based on previous experience to better understand the magnitude of effect sizes.

The graphs in Fig. 7e show the relationship between the sample size (i.e. the number of unique samples per group) and the difference between the upper and lower value of the credibility interval of the estimated interaction term stratified according to the variance and magnitude of the interaction term. The red dots correspond to the estimated interaction terms between treatment arm and time according to variance and sample size. The dashed red line corresponds to the true interaction term.

Greater variance means greater measurement uncertainty. Larger credible intervals mean greater variance and more uncertainty in the estimates, making it more difficult to draw conclusions. Looking at the inflection points of the curves, it can be concluded that data with low variance require fewer samples, 6 in this case, to obtain reliable effect size estimates for any order of magnitude of the effect being measured. If, on the other hand, the data have a higher variance, such as 4, the sample size must be larger (> 15 samples per condition, which in our case corresponds to 5 or 6 mice, considering 4 mammary glands per mouse) to obtain an uncertainty corresponding to that for low variance.

However, we observe that for larger effect sizes (e.g. magnitude = 0.2) one can afford to have around ten samples per set of conditions. In essence, the inherent variability of the measurements and the expected order of magnitude of the effect must be considered in order to determine the appropriate sample size.

We finally calculate the run time in seconds based on sample size and variance (Fig. 7f). The execution time increases linearly as the number of samples in each group increases. In conclusion, as just noted, when designing an experiment, it is important to understand the inherent variability of the data and also to have an idea of the expected effect size estimate in order to be able to establish as correctly as possible the sample size that allows sufficient statistical power for the reliable estimation of the effect to be measured. This clearly requires experience from previous experiments and analyses.

## Discussion

With this work, we show new perspectives for the analysis of longitudinal data using in vivo growth measurements and drug screening data. Our efforts were motivated by the lack of suitable analysis pipelines and algorithms that are easy to use and readily adaptable to the needs of the research community. Our proposed workflow and algorithms are available as an open-source R package (biogrowleR: https://upbri.gitlab.io/biogrowleR/) and has been extensively used by biologists with little to no prior knowledge on longitudinal data analysis.

By using a more sophisticated statistical approach we reduce the number of mice needed for experiments, while gaining more information from the data. Not only does this enhance the integrity of the final data, but reduces unnecessary treatment, an important consideration when undertaking in vivo research, something underscored by the principles of the 3R’s (replacement, reduction, refinement) and recommended by the guidelines for animal experiments [13, 14].

In experiments with inherent variability, such as those utilizing PDX models opposed to cell line models, small effect sizes can still be informative. These measurements are rarely used on their own to draw conclusions, but are interpreted in conjunction with other types of data, such as immunostainings and in vitro experiments. Therefore, it is important to adequately model in vivo growth measurements to understand their dynamics, despite the inherent uncertainty.

There are other approaches to analyze longitudinal data, such as calculating the AUC [15] and repeated-measures ANOVA. However, while these methods focus on the direct interpretation of the *p*-values, they are not the only determinant factor of the experiments. Indeed, study of the Cochrane database for clinical trials shows that the probability of a successful replication of a clinical trial with *p*-value < 0.05 and correct sign, is 40% [16], highlighting the limitations of focusing solely on *p*-values. Bayesian analysis with appropriate priors tailored to the specific experiment and context is recommended [16].

Examining differences in growth rates does not require deriving fold changes, since growth rates are independent of the starting point. This approach does more justice to the inherent variability of biological samples. Hence, when interpreting the results, it is best to consider the probabilities of a specific hypothesis as continuous measurements and not as something dichotomous. This applies for both the frequentist and Bayesian paradigms. Also, we use common levels for the credible intervals as these are routinely used by biologists. Changing these numbers could induce into unnecessary confusion.

Drug screening presents a data modality that cannot be interpreted using the common statistical framework based on the interpretation of *p*-values derived from null hypothesis statistical testing only. As hundreds or thousands of drugs may be tested simultaneously, some of them will be statistically significant, but the effect sizes may be small. This workflow also applies to genetic screens [17] or any other throughput screen that depends on the analysis of multiple conditions simultaneously. Our approach focuses on the direct interpretation of the effect size and its magnitude and direction using Bayesian inference [18, 19], taking experimental uncertainty into account. This may improve data interpretation and improve decisions for downstream experiments.

## Methods

### Hierarchical Modelling

One of the main objectives of statistical inference is to describe the *data generating process*. This is in turn translated into models describing how data were produced and all potential sources of variability that were measured. The most commonly used statistical tool is linear regression, which usually tests the association of a measurement between two groups. Independence between samples is one of the prerequisites when performing these tests. When it comes to longitudinal measurements, such as growth curves, this assumption is no longer valid. This is because repeated measurements for a single sample are dependent and therefore linear regression is not the most suitable tool for this situation.

Hierarchical (non-) linear models (also known as multilevel, mixed effects or random effects) can be used in the presence of such groupings [20, 21]. One example is the multiple measurements of tumor growth from the same mouse [20]. In this case, we can model the outcome using the following formula:

$$\:y\:=\:{\upalpha\:}+\:X{\upbeta\:}+\:Zb\:+\:\epsilon,$$

where y corresponds to the measurement, e.g. luminescence, X is the covariate matrix indicating the time, the treatment from which the measurement originates and their interaction. Z indicates the grouping structure matrix. $\epsilon$ corresponds to the error term and follows a normal distribution with mean 0 and covariance matrix $\:{\Sigma\:}$ of size depending on the number of samples and measurements in each sample. In the case of multiple measurements in a single mouse, it could be specified that the intercept of the linear regression will vary depending on the specific mouse. This is a common example of when starting points are not always the same due to the inherent variability of working with mice. As for the other terms, we will describe them according to the Bayesian framework [2, 18]. The parameters alpha and beta correspond to the global effects that are common across different groups. The parameter b corresponds to the deviations of the global parameters that vary across groups. In short, the probabilistic representation for a y measurement that follows a normal distribution would be:

$$\:y\:\sim\:N\left({\upalpha\:}+X{\upbeta\:}+Zb,{\:{\upsigma\:}}^{2}I\right),$$

where $\:{\upsigma\:}$ corresponds to the variance, and I is the identity matrix.

Within the Bayesian framework, priors are incorporated when obtaining estimated parameters, reflecting what is known prior to data collection. By default, the R *rstanarm* package uses weakly informative priors for the global parameter estimation, which helps stabilize the computation. The weakly informative priors on the intercept and regression coefficients follow a normal distribution centered at 0 with scale 2.5 that still depends on the standard deviation of the covariates and the dependent variable [2]. The default formula for the regression coefficient is:

$$\:{{\upbeta\:}}_{k}\sim N\left(0,\:2.5\times\:\frac{{s}_{y}}{{s}_{x}}\right)$$

where $\:{s}_{x}$ corresponds to the standard deviation of the covariate $\:{x}_{k}$ and

$$\:{s}_{y}=\left\{\begin{array}{cc}sd\left(y\right)&\:\text{i}\text{f}\:\text{f}\text{a}\text{m}\text{i}\text{l}\text{y}=\text{g}\text{a}\text{u}\text{s}\text{s}\text{i}\text{a}\text{n}\left(\text{l}\text{i}\text{n}\text{k}\right)\\\:1&\:\text{o}\text{t}\text{h}\text{e}\text{r}\text{w}\text{i}\text{s}\text{e}.\end{array}\right.$$

For the intercept, covariates are first centered, and the priors are placed on the expected value of y. Thus, the prior would be:

$$\:{\upalpha\:}\sim N\left({m}_{y},2.5{s}_{y}\right),$$

where $\:{m}_{y}$ is the average of the y outcome if the Gaussian family with identity link is used and 0 otherwise. $\:{s}_{y}$ is the same as stated above. For $\:{\upsigma\:}$ and other auxiliary parameters, the default prior is an exponential distribution with rate $\:\frac{1}{{s}_{y}}$. Lastly, we assume $\:b\sim N\left(0,{\Sigma\:}\right)$ as a common prior to $\:b$ [18].

### Simulated Annealing

Simulated annealing is used for combinatorial problems when there is a large search space. Our procedure for randomization represents a special condition of the simulated annealing algorithm. We minimize a metric that depends on the slopes and intercepts of the group subdivisions.

The rule used is based on the differences between the intercepts and the differences between the slopes. For example, suppose we want to divide our data into two groups. The initial splitting is randomized and timepoints are shifted such that the maximum value is 0. By doing so, the intercept of the curves corresponds to the average of the last measurement. The slopes and intercepts of the linear regression are obtained and the pairwise differences between the slopes and intercepts are calculated as follows:

$$\:\text{D}\text{i}\text{f}\text{f}\text{e}\text{r}\text{e}\text{n}\text{c}\text{e}\:\text{s}\text{l}\text{o}\text{p}\text{e}\text{s}= \sum\limits_{i,j=1;i < j}^{n}\left({s}_{i}-{s}_{j}\right),$$

where $\:{s}_{i}$ and $\:{s}_{j}$ are the slopes for group $\:i$ and $\:j$ and $\:n$ is the total number of groups. Similarly, the pairwise difference of the intercepts is defined by

$$\:\text{D}\text{i}\text{f}\text{f}\text{e}\text{r}\text{e}\text{n}\text{c}\text{e}\:\text{i}\text{n}\text{t}\text{e}\text{r}\text{c}\text{e}\text{p}\text{t}\text{s}=\sum\limits_{i,j=1;i < j}^{n}\left({{\upgamma\:}}_{i}-{{\upgamma\:}}_{j}\right),$$

where the $\:{{\upgamma\:}}_{i,j}$ corresponds to the intercept of the linear regression fit. To accept the new split the sum of pairwise differences of slopes and the sum of pairwise differences of intercepts needs to be smaller than in the previous split. If the new split is not better than the previous one, we regenerate a split based on the *temperature* and *energy* over time. This means that if the new split is not better and the algorithm is in its first iterations, there is a high probability that the new split will be used. This allows the algorithm to further explore the search space and avoid local minima. To be more precise, if the condition to switch samples from one group to another fails, then we calculate a value, i.e. the cost to move, which will be used to decide whether the new split is maintained or not:

$$\:\text{C}\text{o}\text{s}\text{t}\:\text{t}\text{o}\:\text{m}\text{o}\text{v}\text{e}=\frac{{\sum}_{i,j=1;i < j}^{n}\left({{\upgamma\:}}_{i}^{new}-{{\upgamma\:}}_{j}^{new}\right)\:-\:{\sum}_{i,j=1;i < j}^{n}\left({{\upgamma\:}}_{i}^{old}-{{\upgamma\:}}_{j}^{old}\right)}{{\sum}_{i,j=1;i < j}^{n}\left({{\upgamma\:}}_{i}^{new}-{{\upgamma\:}}_{j}^{new}\right)},$$

The energy is then calculated as:

$$\:\text{e}\text{n}\text{e}\text{r}\text{g}\text{y}={e}^{\left(-\left(\text{c}\text{o}\text{s}\text{t}\:\text{t}\text{o}\:\text{m}\text{o}\text{v}\text{e}\right)/\text{t}\text{e}\text{m}\text{p}\text{e}\text{r}\text{a}\text{t}\text{u}\text{r}\text{e}\right))},$$

where temperature is defined iteratively as:

$$\:\text{t}\text{e}\text{m}\text{p}\text{e}\text{r}\text{a}\text{t}\text{u}\text{r}\text{e}\:=\:\text{t}\text{e}\text{m}\text{p}\text{e}\text{r}\text{a}\text{t}\text{u}\text{r}\text{e}\times\:{\upalpha\:},$$

with $\:{\upalpha\:}$ set to 0.5 by default and the initial temperature is 1. Then, we sample a value from a uniform distribution from 0 to 1 and if the sample is below the energy value the new split is accepted. With the temperature decreasing over time, energy also decreases, making a new split that was rejected based on the metric more difficult to be accepted.

### Likelihood Ratio Test

For comparing growth curves between treatments using the frequentist approach, the likelihood ratio test is used. The full model is specified with an interaction between the time and the treatment group (for two different groups, pairwise). The restricted model is specified without the interaction term. Since we are using hierarchical modeling (mixed effects model) and the *lme4* package, *p*-values are not readily available. Therefore, we test for differences between groups as differences between slopes, which represent growth rate. The *lrtest* function from the *lmtest* package calculates the likelihood ratio test for an unrestricted model and a restricted model. The test statistic is then compared to a Chi-squared distribution that depends on the number of parameters for both models.

### Visualization Tools

The R packages *tidybayes* (v3.0.2) [22] and *ggdist* (v3.1.1) [23] were used to create dotplots, uncertainty interval and forest plots. They accept *rstanarm* [18] and *brms* [19] objects for plotting. biogrowleR accepts outputs from both packages. For other plots, *ggplot2* (v3.4.2) from *tidyverse* (v1.3.1) [24] was used in R 4.2.0.

### Drug Screening

Cell lines were obtained from ATCC and maintained at 37 °C in humidified incubator in an atmosphere of 5% CO2. T47D:*RFP-Luc2* cells were cultured as described [9] and plated at 5 × 10E3 cells/well of a 96-well-plate. Cells were treated with compounds dissolved in DMSO to final concentrations in table below, and imaged (brightfield and red fluorescence) at 6 h intervals using an Incucyte SX3 (Sartorius) for up to 4 days.

**Table:** 

Compound	Concentration
DMSO	0.5%
17-β-estradiol (E2)	10 nM
Progesterone (P4)	10 nM
Dihydrotestosterone (DHT)	10 nM
Hydrocortisone	1 µM
Mifepristone	1 µM
Fulvestrant	100 nM
ARV471	100 nM
PR PROTAC (1230)	100 nM
ARV110	30 nM
Abemaciclib	100 nM
Palbociclib	100 nM
Olaparib	1 µM

### R Package

This project is available as an open source package in R (https://upbri.gitlab.io/biogrowleR/; https://gitlab.com/upbri/biogrowleR/).

## Data Availability

No datasets were generated or analysed during the current study.
